# A topic modeling approach for analyzing and categorizing electronic healthcare documents in Afaan Oromo without label information

**DOI:** 10.1038/s41598-024-83743-3

**Published:** 2024-12-30

**Authors:** Etana Fikadu Dinsa, Mrinal Das, Teklu Urgessa Abebe

**Affiliations:** 1 Department of Computer Science and Engineering, Engineering and Technology, Wollega University, Oromia, Ethiopia; 2 Department of Data Science, Indian Institute of Technology Palakkad(IIT Palakkad), Palakkad, India; 3 Department of CSE, Adama Science and Technology University, Oromia, Ethiopia

**Keywords:** Afaan Oromo, Topic modeling, Latent dirichlet allocation, Text analysis, Information retrieval, Classification, Computer science, Software

## Abstract

Afaan Oromo is a resource-scarce language with limited tools developed for its processing, posing significant challenges for natural language tasks. The tools designed for English do not work efficiently for Afaan Oromo due to the linguistic differences and lack of well-structured resources. To address this challenge, this work proposes a topic modeling framework for unstructured health-related documents in Afaan Oromo using latent dirichlet allocation (LDA) algorithms. All collected documents lack label information, which poses significant challenges for categorizing the documents and applying the supervised learning methods. So, we utilize the LDA model since it offers solutions to this problem by allowing discovery of the latent topics of the documents without requiring the predefined labels. The model takes a word dictionary to extract hidden topics by evaluating word patterns and distributions across the dataset. Then it extracts the most relevant document topics and generates weight values for each word in the documents per topic. Next, we classify the topics using the represented keyword as input and assign class labels based on human evaluations topic coherence. This model could be applied to classifying medical documents and used to find specialists who best suitable for patients’ requests from the obtained information. As a conclusion of our findings, the topic modeling using LDA gave the promised value of 79.17% accuracy and 79.66% F1 score for test documents of the dataset.

## Introduction

Afaan Oromo (often known as Oromo) is the lowland east Cushitic linguistic branch spoken largely in Ethiopia and neighboring countries, such as Kenya, Tanzania, Sudan, Djibouti, and Somalia^1^. The number of speakers of Afaan Oromo in Ethiopia is expected to be above 40 million (one-third of Ethiopian populations)^2^. It is broadly used in religious, commercial, political, social, education, health sectors, and in the mass media. Afaan Oromo has a writing system called “Qubee”, which is a Latin-based orthography^3^. Afaan Oromo, having its own completely unique grammatical structure, syntax, script, ways of pronunciation, and sentence length, which have an impact on textual document analysis, filtering, and finding representative topics of the collection of documents is a big challenge^4–6^. As Afaan Oromo is new in the field of natural language processing (NLP), therefore less work is done in the literature. Today’s is the era of hospital digitization, so it is required to capture patient information^7,8^. Currently, the majority of health-related information available is represented as text documents^9^. The huge amount of electronic text is available in health organizations is quickly growing because of the overflow of disease information, patient records, and related documents^10^. This presents substantial challenges of extracting relevant knowledge and retrieving information when it is required. Due to this the development of efficient tools or strategies for indexing, searching, and organizing massive amounts of data has become a necessity^11–13^. However, it is more challenging in the medical domain than that of other domains since the electronic medical documents are characterized by high-dimensionality and sparsity. We may find many medical characteristics, such as having abbreviations, complex terminology, borrowed terms, and phrases that are specific to this field, which produces the issue of the high-dimensionality, and this makes analyzing and processing the medical data more challenging^14^.

Topic modeling is the strategy of extracting the hidden topic inside the documents, as well as processing the text data and categorizing it automatically^15,16^. It is type of statistical models and method of text mining which used to organize bulky collections of textual documents into smaller number of “topics”. Topic modeling has gained a lot of focus in unsupervised learning in the past several years^17,18^. It is used to obtain the topics of several news stories from online news portals, and it can be described as discovering groups of words (topics) from textual documents that are able to well represent the information about the documents. Since Afaan Oromo is a low-resource language, leveraging deep neural networks for analyzing unstructured healthcare data is challenging due to the insufficiency of annotated labeled data and pre-trained models. The deep learning models typically require extensive datasets and computational resources to implement effectively. Consequently, we have opted to employ the topic modeling techniques from ground up. This methodology allows us to uncover hidden or latent topics and patterns within the collections of documents without the need for huge labeled datasets.

This work proposes an approach that focuses on topic modeling development for unstructured electronic health documents written in Afaan Oromo using the LDA model. Tools and models developed for other languages cannot work for Afaan Oromo because language structure and syntax is totally different. As far as our knowledge no work has been accomplished on topic modeling for this language in this domain. To tackle this gap, our goal is to apply LDA algorithms to extract latent topics from unstructured documents and use the extracted topics and associated documents as further input for information retrieval and categorization purposes. This technique is more reasonable and practical for low-resource languages like Afaan Oromo, where computational resources and data availability are limited. By utilizing topic modeling, we aim to improve the understanding of healthcare document issues and trends in the Afaan Oromo-speaking community, providing groundwork for future research and potential applications in healthcare document analysis and information retrieval.

The major contribution of this work summarized as:

- We prepare an unstructured corpus from healthcare document related domains in Afaan Oromo.
- We propose latent dirichlet allocation for the purpose of categorizing documents without label information.
- Through the experiments on our datasets, we demonstrate the capability of our model in simultaneously extracting topics and categorizing documents.
- From the current experiment, the proposed LDA model gives the promised results for categorizing healthcare documents.

This paper is separated into subsections. The second section introduces the background of topic modeling. Section three discusses related studies. Section four presents the dataset preparation. Section five presents proposed topic modeling techniques. Section six illustrates the experiment and result. The discussions are provided in section seven. In section eight, we draw conclusions and recommendations for further research.

## Background of topic modeling approaches

Topic modeling (also known as topic detection, topic extraction, or topic analysis) is statistical practice with a collection of algorithms for discovering, revealing, extracting, and annotating secret structure of data from a massive of collections of document^14,19^. In natural language processing, topic detection is performed by using two different methods: supervised and unsupervised techniques. In the first technique, we know the topic labels in advance, and we want to categorize the documents, assigning one or more topics per document. In the second technique, we are trying to automatically extract topics from the documents using collections of words that are most representative of the content. The words or terms that describe each topic are somehow inaccurate and only suggestive of the overall contents. Another typical problem in this field is detecting the optimal number of topics in a totally automatic way^20^. Topic models represent three entities: collections, constructs, and topics^21^. In a textual document, constructs are the terms that comes together to create a collection. Generally constructs are words that are clustered to establish a document. The topic is a bunch of constructs that come together to describe the semantic meanings. It is an ideal view of documents that is as pure as possible. It is also an identical collection of constructs that have semantically so many similarities. Mathematically, topics are defined as the probability distribution over their constructs^22,23^.

There are different types of topic modeling algorithms used for finding topics from large collection of textual documents. Some of them are latent semantic analysis (LSA)^14^, probabilistic latent semantic analysis (PLSA)^24^, non-negative matrix factorization (NMF)^15^, and latent dirichlet al.location (LDA)^24^. The LSA is a technique which employs statistical methods and mathematical computations useful for huge text datasets to extract and signify contextual meaning of the words^14,25^. It uses particular value decompositions to decrease the high-dimensionality of the vector using the TF-IDF scheme. However, it lacks a strong statistical foundation and suffers from high mathematical complexity^26^. The PLSA is the other statistical scheme for analyzing text documents based on hidden class model. PLSA has concrete statistical foundations, and it can discover hidden topics and return better performance when related to LSA. The NMF uses matrix factorization which is non-probabilistic and it belongs to a cluster of linear algebraic procedures. It works on the TF-IDF, converting data by splitting down the matrix into smaller ranking matrices^15,27^. The Pachinko Allocation Model (PAM) is another newest topic modeling techniques that employs a hierarchical structure and captures dependencies among topics to represent more complex topic relationships and correlations^19,28^. The LDA is the probabilistic topic model and most popular mechanism used for latent topic extraction by assuming independence between topics. This method is unsupervised method for topic detection based on latent variables or a hidden topic model. It has been broadly developed in research on studying a textual document topic^29,30^.

**Fig. 1:**
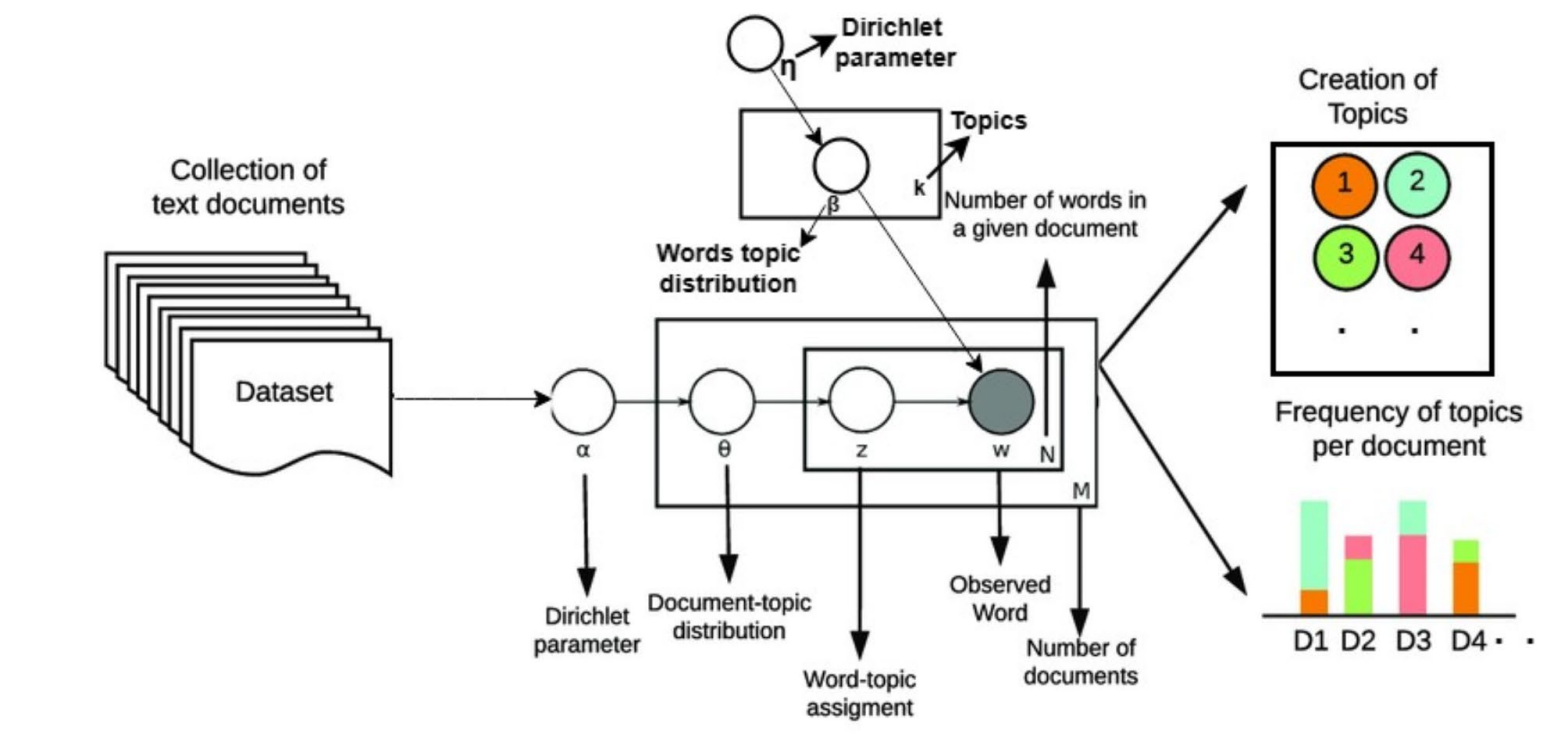
The general graphical representation of latent dirichlet allocation Model (LDA).

In our methodology, LDA was selected for its computational efficiency and ability to create interpretable topics without complex hardware or deep pre-trained embeddings since it could be used in low-resource languages. Since, LDA is probabilistic by nature and works with smaller datasets on limited computing power, unlike the rather resource-intensive BERTopic using transformer models^30,31^. Likewise, NMF may struggle with sparse or unstructured data and generally requires thorough preprocessing; however, it may be useful in certain cases^30^. Low-resource situations benefit greatly from LDA’s uncomplicated implementation, computational efficiency, and robust performance, as well as its capacity to extract latent topic structures through word co-occurrence patterns. Additionally, the LDA is used MCMC (Markov chain Monte Carlo) technique to infer topics, where NMF is another alternative but MCMC has been observed to perform well on sparse and small datasets compared to NMF. The LDA architecture utilized for topic modeling, as illustrated in Fig. 1. In this model every node is a random variable, and the observed variables (*W*) are shaded. *M* refers to the total number of documents. Alpha *(α)* is dirichlet parameters. The *θm* represents the topic proportion per-document. *Zm*,* n* indicates the topic assignment per-word. *Wm*,* n* is an observed word. Different topics are denoted by K. *N* represents the number of terms in a given document. The probability distribution of the top words for a particular given topic K is denoted by the symbol *βk*. Topic hyper-parameter is represented by Eta *(η)*. Generally, the LDA model finds the hidden (latent) topics and subjects in a given corpus, and an observed variable is word^23^.

## Literature review

Topic modeling is a way to automatically extract topics within an unstructured document item and mine hidden patterns from a given corpus of text. As a result, making decisions becomes simpler. In this experiment, we used a popular topic modeling procedure known as latent dirichlet al.location^23^ to extract the hidden topics and categorize the documents. To the best of our knowledge, this study is the first attempt to utilize topic modeling techniques using LDA for the classification of unstructured Afaan Oromo electronic medical documents.

Li-xia Luo^32^, proposed the combination of the LDA model and the convolutional neural networks (CNN) to increase the performance of internet public sentiment analysis methods. First, they collected review documents from the network for pre-processing. Then, they utilized the LDA model to train a latent semantic topic word distribution for the documents; the topic distribution is constructed from short text feature vector representation. Finally, the CNN-GRU is applied as a classifier. According to the contribution feature matrix, the CNN-GRU supports the association between text and text, words and words, to achieve the most accurate review text categorization. The experimental results display that their method can efficiently advance the accuracy of the review classification. In paper^33^, it is proposed to combine the LDA model with multi-class and logistic regression for the topic modeling multi-step classification method in order to find and extract to classify topics from the unseen text documents without depending on human labeling. They use domain expert explanation in order to accurately detect clusters of terms according to a particular topic. Their findings propose that the two processes were balancing in terms of detecting textual topics and overcoming challenges of understanding the array of topics in the LDA output.

Thielmann et al.^34^illustrate the combination of Support Vector Machines (SVM) and the LDA topic modeling as a multi-classification approach that avoids human labeling issues. The findings specify that unsupervised textual document classification implemented using web scraping, one-class SVM, and LDA modeling produces very precise classification results for a range of data sets. However, these approaches also need the assistance of the domain expert to detect the LDA output topics retrieved. The paper^16^, is exploring text extraction, mining, and analyzing useful information from unstructured textual documents using sentiment analysis and LDA topic modeling by examining the English twitter text documents. These approaches allow the people to discovery data more efficiently and effectively. Both sentiment analysis and the LDA topic model can also be useful to offer insight into views in scientific and business fields.

The work^35^, proposed a model that extracts topics from short texts, specifically Twitter plain texts. The key features of their proposals are the use of word embedding for topic modeling techniques and k-means for clustering semi-automatic explanations of the tweets. Also, their model surpasses the difficulties of sparseness and word ambiguity in the text documents by using the word-embedding methods. The author^36^ performs an evaluation of internal indexes of topic modeling and document clustering techniques over two health-related Twitter datasets. The results show that Online Gibbs LDA and Twitter LDA get better performance for finding topics and grouping tweets. They provide health experts with this comparison result to select the most appropriate application for their tasks.

C.Moody^37^proposed a modified LDA-to-vector method that is a mixture of the word-to-vector and the LDA model. They trained the models using the 20 newsgroup data and the hacker’s comments corpus. As an experimental result, this method was efficient and simple to utilize in the automatic classification framework, and it can lead to the unsupervised illustrations that are simpler to interpret. The paper^38^, proposes legal information extractions from an Indian judicial dataset. They used three diverse topic modeling techniques: LSA, NMF, and LDA, and found that the LDA outperforms the other applied topic modeling methods based on experimental results. Their objective is to obtain the legally hidden information from the court decisions and utilize it to build labeled data from various legal applications. After they applied the LDA model to the dataset, the labeled legal ruling dataset for the Indian judges system was generated.

## Afaan Oromo dataset preparation

This section gives a short overview of the dataset used for the current works.

A. **Data Collection**.

Data assortment is the most exhausting tasks in this research field, especially in low-resource languages. To build our noble corpus, we used electronic healthcare documents, which were documented in Afaan Oromo. Our final dataset consists of 3000 documents on health-related issues. All collected documents lack label information, making it challenging to classify or categorize them perfectly. This lack of labels poses significant challenges for organizing the documents and applying supervised learning techniques that rely on labeled datasets. This dataset is gathered from healthcare sector related patient symptoms and disease information. For instance, the sample document from the Afaan Oromo Healthcare document (AOHD) is presented in Table 1.

**Table 1:** Sample data from the Afaan Oromo Healthcare document (AOHD) corpus.

S.no	Documents
1	karaa bobbaa koo dhiigni na ba’a, keessisaa na guba, hooqi hooqii na godha, bobbaan dafee naaf hin ba’u
2	mataa keessaan dhiita’ee ba’e, bakki sun diimatee ni guba
3	Dhangala’aa karaa qaama koon na ba’a, foolii badaa kan qabuu fi adii kan ta’etu na ba’a, shiffi jedhee natti bibbiqila
4	Mataa koo na bowwaafata, yaada natti baay’isa, Irribni sirnaan naaf hin dhufu, nan yaadda’a
5	Jilba koo keessa deeme na nyaataa, yeroo dheeraa deemu hin danda’u

All collected AOHD’s are stored as texts of Afaan Oromo sentences. In this experimentation, the focus is on textual-based medical data, where all applicable information is provided in written form. The details the demographic information’s and other relevant attributes are encoded within the text itself, guaranteeing that the corpus covers the necessary contextual information’s for analysis. They all can be treated as strings or text documents. However, we cannot provide this raw data directly to the model. So, we have employed the pre-processing technique to make the datasets more suitable for the model.

B. **Data Preprocessing**.

Text data with Afaan Oromo language properties is used as input data in the current experiment. Before using any machine learning algorithm, this text data must be preprocessed or cleaned. Once the dataset is ready, we apply common preprocessing steps like tokenization, punctuation removal, lowering case, stop word removal, and removal of words with less than three characters. The dataset used in this experiment focuses on an electronic health document written in Afaan Oromo.

In the current study, after the completion of pre-processing procedures, the necessary topic model libraries and their dependencies were loaded in Python in order to perform the LDA models for the dataset to identify the topics and keywords. Then, the optimal number of keyword(K) is determined for the corpus. The LDA models begins with random assignment of topics to each keyword and iteratively improves the assignment of topics to words in the documents^24,39^.

### 1) Tokenization

The document is broken down into the sentence, and the sentences are broken down into the words or tokens. We synthesize words from a continuous stream of text. In the case of our experiment, we used text split methods since words in the Afaan Oromo languages are separated by blanks (white space, commas, quotations, semicolons, and periods) are frequently used. Then it can be converted to single words (noun, pronoun, article, verb, preposition, conjunction, punctuation, alphanumeric, and numerals) without considering their relationships or meanings.

### 2) Lowering all characters

Text analysis is case-sensitive. In a probabilistic model, the frequency of each letter in the sentence is counted, and lowercase and uppercase are considered as different letters. For instance, the words “dhukkuba” and “DHUKKUBA” in Afaan Oromo are the same. But the model will treat as dissimilar words if the case conversion is not applied before used. So, all the letters should be converted to lowercase.

### 3) Remove stop-words

In particular, stop-words can be found in any textual document. Because of this, traditional topic models cannot acquire accurate topics from documents containing stop-words, irrespective of the actual semantics of the topic. This can also lead to low topic interpretability. To improve the topic’s interpretability, we omit words that are less important and terms that are most frequently used in a language.

**4) Remove special characters**: special characters like, ([], [,], [:], [.], [”], [;], [/], [˜], [!], [[]], [@], [#], [%], [ˆ], [$], [*], [&],[(], [)]) will be removed, and we Preserve the single quote (’) for Afaan Oromo.

C. **Dictionary Creation**.

To apply LDA algorithms, we utilize bag of words (BOW) corpus. For this purpose, the concept of the gensim dictionary is used. Gensim is designed to find semantic topics in text documents and manage large text collections^40^. It takes the tokens, or words, to be changed into their separate IDs. This provides the dictionary object features, which map each token to have a distinct integer-ID. These are achieved by converting the textual document into a word list and then sending to the corpus. Dictionary () object, a list of textual documents is used to produce a BOW corpus. Then a tokenized list of terms is provided that the Dictionary.doc2bow () objects, then generates the word matrixes, and that matrix is supplied as the model’s input.

## Proposed topic modeling approaches for Afaan Oromo health documents

**Fig. 2:**
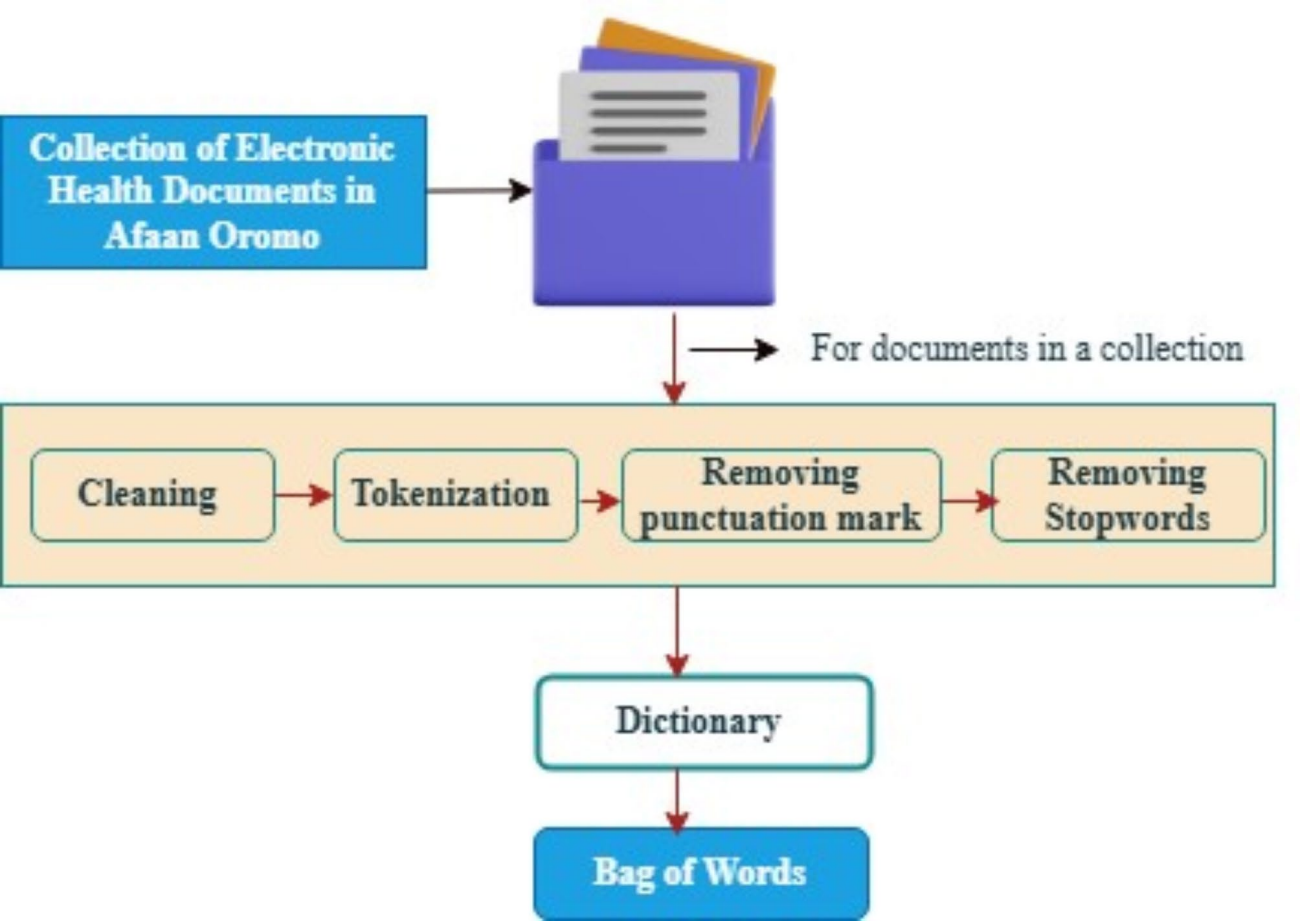
AOHD preprocessing architectural diagram.

**Fig. 3:**
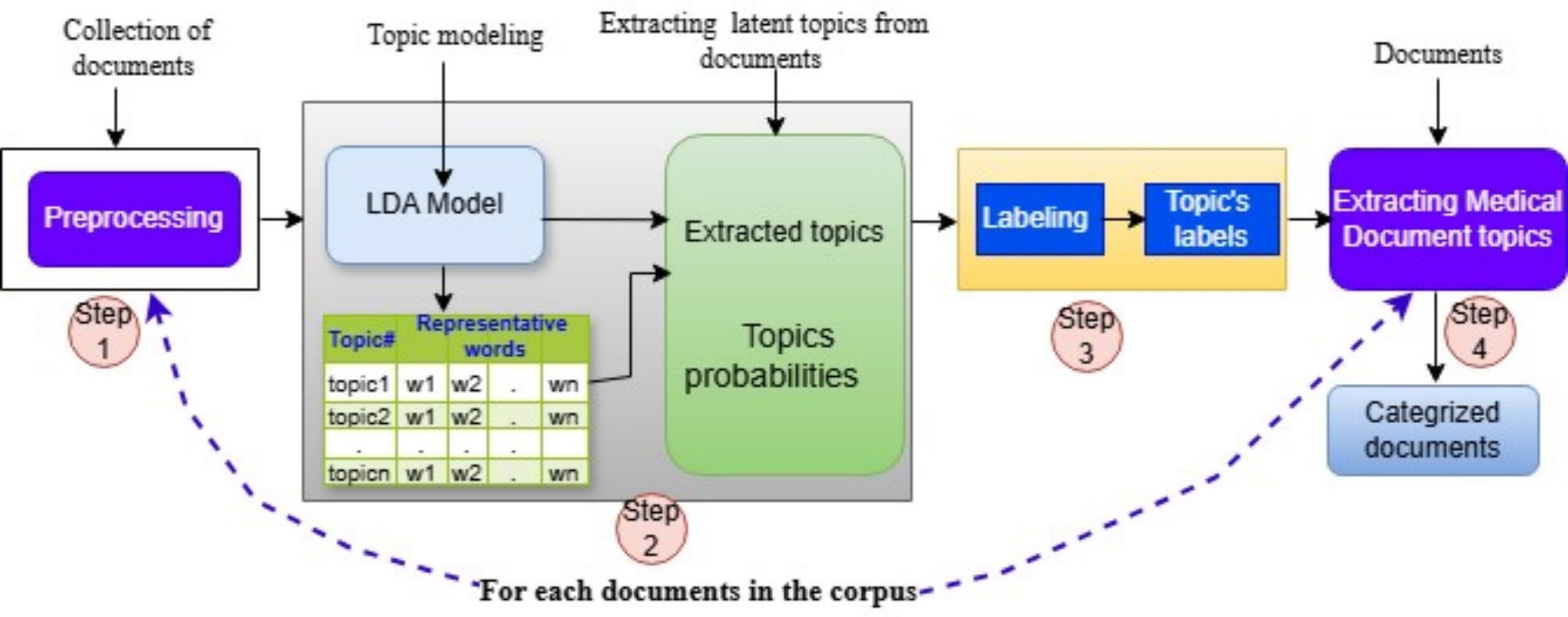
Workflow of the proposed topic modeling methodologies utilizing LDA.

The proposed model uses LDA for creating labeled documents through latent topic extraction, as it is suitable for further classification of unseen documents. Our system architecture can be separated into 5 different components. This can be presented in Figs. 2 and 3. The components are: (1) Data pre-processing; (2) Dictionary creation; (3) LDA model; (4) Labeling topics; and (5) save the labeled documents.

## Experiment and result

### Training topic model with Afaan Oromo health documents

The aim of topic modeling is used to mine the unstructured documents’ main topics^17^. In our methodology, we used LDA, which is the generative probabilistic model and is a popular topic modeling technique, for discrete data sets such as text corpora^23,30,41^. The LDA will receipts the pre-processed texts as input. Then group the words inside the text documents based on most similar meanings^42^. The words in the topics are associated with conditional probability and representing a confidence degree, which means how much each word or terms is relevant to that cluster’s topics. The obtained groups of words signify the diverse topics in the document. The main difficulties of using LDA model is that it does not label the extracted topics as mentioned in^35,43^. To solve this issue, we manually label this group of words in the themes. In our work, we limit the top 10 words and 10 topics. When using human judges to interpret the topic model results by domain experts, the best effective technique is to carefully analyze the underlying keywords of the model output. Thus, the issue of unlabeled extracted topics is resolved. After labeling the acquired clusters of words, extract the most representative keywords of each topic and each documents. We initially, we extract a topic matrix vs. document. In this matrix, every term is associated with the probability of belonging to a specific topic.

The following summary of steps carried out during LDA model training steps.

1. To implement the topic detection, processing of the topic keyword distribution, and the topic document distribution, the Gensim package multicore LDA models is used.
2. The best number of topics we used by running the LDA model on our corpus is 10, and for each topic we used 10 keywords.
3. The final step assigns the topics to each document using the model by finding the probability distribution of the sentence per generated topic assignment.

Finally, we obtain the topics of the documents and, for each topic, assigned the label along with the most illustrative words associated with those documents.

A. **Dominant topic extraction and output visualization**.

Table 2 reports some topics that are extracted from the electronic health document. As observed from this experiment, every topic consists of 10 keywords associated to the topics. Each extracted word in each topic has a correspondent probability ratio. We used 10 most probable keywords for each topic. However, there are a few words that might have nonsense to those topics, but most of them are relevant to associated topics. Some of the topics are a little mixed, and meaning can be dissimilar, but most of the other extracted topics give sense, and they can be clustered as a category in the document. To this achieve this optimal topic and keyword selection, we relied on various model evaluation metrics, namely coherence values for topic interpretability assessment and perplexity scores for measuring model ability to predict data. The evaluation of these metrics was conducted iteratively by varying the number of topics and observing their impacts on model performance. This assortment procedure was guided by both quantitative metrics and qualitative assessments by the domain experts to ensure transparency and relevance. Each keyword is listed based on its probability distribution in descending order. The meanings of each keyword are provided in English in the context of medical concepts.

**Table 2:** Extracted topics from AOHD dataset when LDA model applied.

Topic	Top 10 topics Keywords
Topic 0:	Dhaqna(body), dhufee(came), gogsa(dries up), roqomsiisa(rocking), si’a(times), dhabne(lost), dadhaba(tiredness), dugda(back), barbade(want), qorachiisu (test)
Topic 1:	Ulfaa(pregnant), hordoffi(follow-up), fuula(face), laguu(menstruation), seeran(legally), dhiigatu(bleeding), darbee(occasionally), dhiita’ee(swollen), garmalee(excessively), baayyata(to increase)
Topic 2:	Dhufe(came), mataa(head), afuura (breath), cabe(broken), ilkaantu(teeth), kuta(cut), mala(the method), gurra(ear), dhufe(come), isaan(they)
Topic 3:	Ija(eye), dhukkuba(pain), gubaa(burns), argu(to see), jaamsee(blinded), laphee(chest), keessa(in), dhaqna(body), walitti, huuba
Topic 4:	Keessa(in), mataa(head), waraana(stambs), gurra(ear), dugda(back), nyaata(eats), garaa(stomach), gadi(below), ba’e(out), dhiita’ee(swollen)
Topic 5:	Afaan(mouth), nyaata(food), laga, deebisa(vomit), keessa(in), hadheessa(bitter), guba(burns), goga(dry), baay’ee(several), joonjeessa(confuse)
Topic 6:	Cabe(broken), miilla(feet), harka(hand), dhiiga(blood), irratti(upon), kufeen(fell), barbaade(want), qufaasisa(coughing), ba’e (out), qufaa(qufaa)
Topic 7:	Garaa(stomach), guba(burns), hooksisa(hooks), kaasa, amma(now), nyaata(eat), afaan(mouth), addeessa, gogaa(skin), irra(on)
Topic 8:	Dide(refused), qaama(body), cabe(broken), kufee(fell), miilla(feet), lafee(boone), ba’e(out), jaame(blind), dhowwaa(forbid), saala (sexual organs)
Topic 9:	Dhukkuba(pain), ilkaan(teeth), keessa(in), nyaata(eats), cabe(broken), dhiita’ee(swollen), keessaa, ijatu(eyes), guba(burns), morma(neck)

**Fig. 4:**
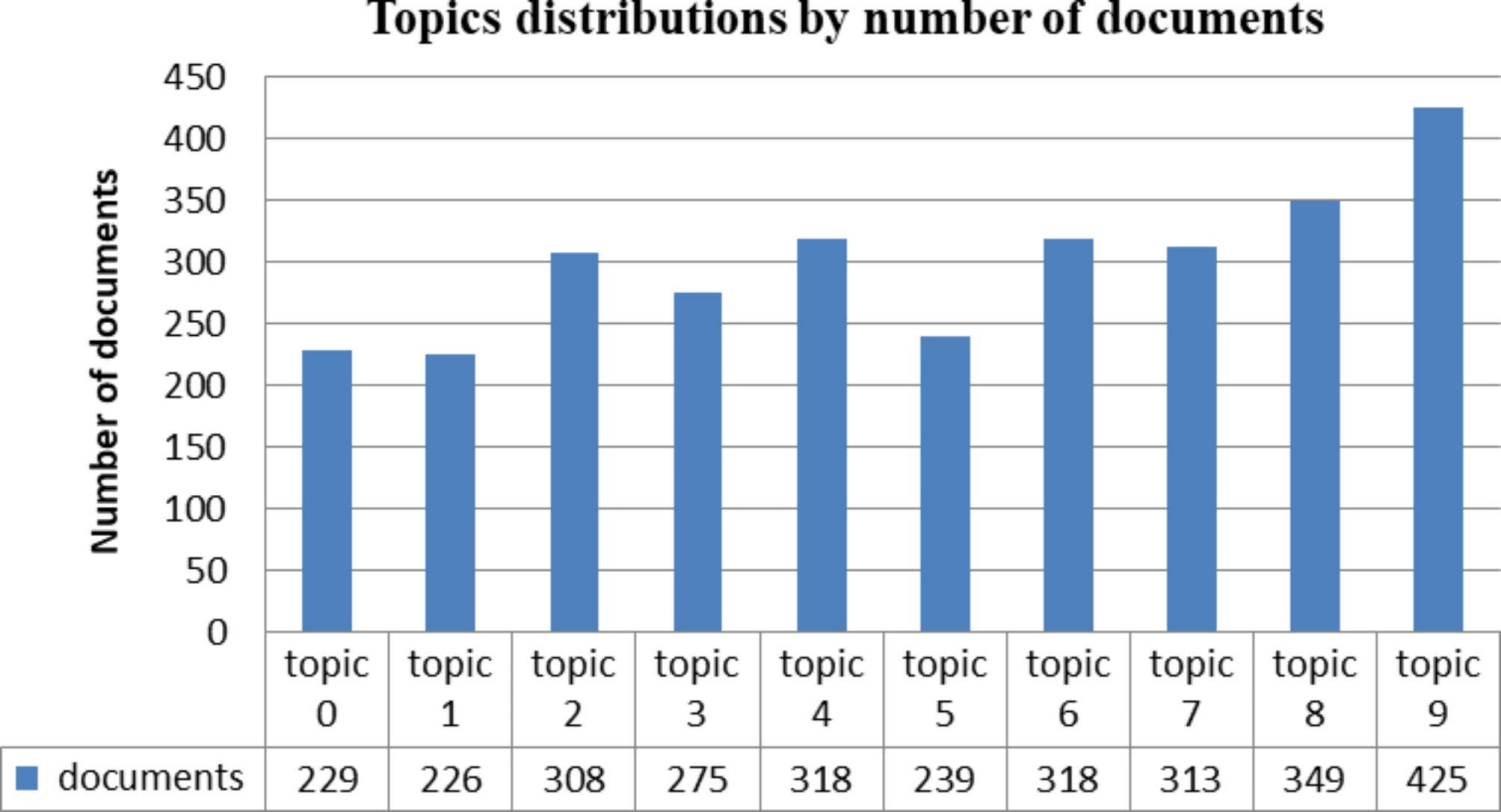
Topic distributions of documents in corpus of the AOHD dataset.

As we stated in the training topic modeling section, every text document in corpus will be assigned to one theme class based on a high probability contribution to the dominant topic. After training the LDA models, we present the distribution of the topics in the document for each sentence and the weight of the topic and the keywords in a pleasantly formatted output, and the sample results were as shown in Table 3. Figure 4 illustrates that document topic distribution weights for all documents in our corpus.

**Table 3:** Sample document topic distribution after implementing LDA model from the AOHD dataset.

ID	Dominant topic	Contributions	Keywords	Cleaned text
1	5	0.7270	Afaan, nyaata, laga, deebisa, keessa, hadheessa, guba, goga, baay’ee, joonjeessa	garaa keessa uursa ciniina mataa ulfaata
2	2	0.4199	dhufe, mataa, afuura, cabe, ilkaantu, kuta, mala, gurra, dhufe, isaan	mataa dhukkuba matatu nanna’a
3	9	0.4229	Dhukkuba, ilkaan, keessa, nyaata, cabe, dhiita’ee, keessaa, ijatu, guba, morma	ilkaantu dhukkuba ho’a qabbana’a fayyadamu dhahata
4	7	0.8199	Garaa, guba, hooksisa, kaasa, amma, nyaata, afaan, addeesa, dhowwe	dhagna bakkeerra addeessa hooksisa
5	1	0.9000	Ulfaa, hordoffi, fuula, laguu, seeran, dhiigatu, darbee, dhiita’ee, garmalee, baayyata	laguu seeran dhufu ji’aa darbee dhufa jeeqamaadha

B. **Topic labeling**.

In this experiment, topic modeling produces word distribution that gives meaningful topics similar to word topic probability distribution according to the chosen number of topics. Each word has a probability of each document and the relevance of one topic to the other. After the LDA model has been generated, a document can be determined based on the distribution of topics describing the document’s collection of terms. The LDA model uses a BOWs assumption, which means the order of words is ignored in the document. From our experimental result, the generated topics with important keywords are listed in Table 2. Each term under each topic or theme is assigned based on a weight, with the highest ranking words having greater weights.

Defining the core meaning of these extracted topics is crucial. However, the model does not tag topic labels automatically. The labels are made manually by carefully observing the grouped words in each topic, since LDA will only return the group of keywords known as topics. Despite the developments in statistical methods, the interpretability of the model output is not assured because of the difficulty in the language^44^. Therefore, when merging the word matrix of each topic, it is required to assign the artificial labels to correctly reflect the internal association and context of corresponding keywords. To label the extracted topics we involved three domain expert annotators. The labeling process was guided by balancing interpretability and coherence values to ensure accurate and meaningful labels. When disagreements arose among the annotators, particularly regarding ambiguous words such as homonyms or polysemy, we took the majority to resolve it. This procedure expected to maintain consistency and strengthen the interpretability of the extracted topics in the medical context. Based on the extracted topics was labeled and with their distribution results so that we could easily describe each topic. In Topic 0 most keywords are “dhagna”, “dhufee”, “gogsa”, “roqomsiisa”, “dugda”, “qorachiisu” are showing that this topic is about nervous related disease, the most common words in Topic 1 “ulfaa”, “hordoffi”, “laguu”, “dhiigatu”, “darbee”, “garmalee”, and “baayyata” are implying that the topic is about gynecology, Topic 2 is mental illness, Topic 3 is eye disease, Topic 4 is ear disease and disorders, Topic 5 is internal disease, Topic 6 is chronic disease, Topic 7 is skin disease, Topic 8 is orthopedic conditions, and Topic 9 is dental related disease are presented in Table 4.

**Table 4:** Manually formed labels for the extracted keywords per topics.

Topic numbers	The titles of the topic
Topic 0	Nervous disease
Topic 1	Gynecology
Topic 2	Mental illness
Topic 3	Eye disease
Topic 4	Ear disease and disorders
Topic 5	Internal disease
Topic 6	Chronic disease
Topic 7	Skin disease
Topic 8	Orthopedic conditions
Topic 9	Dental disease

C. **Topic model evaluation**.

To evaluate the output of the model we used the two qualitative metrics the coherence value (Cv) score from topic coherence framework and the quantitative method perplexity^39,40^.

**Perplexity**.

It is a standard quantitative method to evaluate topic models. It is termed as how successful our model predicts the sample word probability over the documents based on term occurrences in the topics. Perplexity^45^ is defined as algebraically corresponding to the inverse of the geometric mean per word probability and calculated for the corpus D test by computing the natural exponent of the mean of log-likelihood of the corpus of words, as presented in Eq. 1. In topic modeling, the lower perplexity value shows the better generalization ability.

1 $$\:\text{P}\text{e}\text{r}\text{p}\text{l}\text{e}\text{x}\text{i}\text{t}\text{y}\left(\text{D}test\right)=exp(-\frac{{\sum\:}_{d=1}^{m}\:log\:p\left(wd\right)}{{\sum\:}_{d=1}^{m}\:Nd}\:\:)$$

Where, D*test* is the test dataset, M is the number of textual documents in the test datasets, wd represents the words in document d, and p(w, d) is the likelihood of the document d. The perplexity of our LDA model at topic 10 based on the experiment is −6.403. We used perplexity to evaluate the model result for guessing the number of topic k for the model assessment of our dataset since the model assumed the K parameters are mandatory. Calculating log perplexity yields a negative value due to the logarithm of a number.

**Coherence value**.

In topic modeling, the coherence value is the score measure of semantic likeness between each word in the topics extracted^17^. When topics generated are semantically interpretable, topic coherence scores are high. Several measures based on the word co-occurrence score of the most significant terms for each distinct topic have been applied in the topic extraction literature. The best practice to determine how a topic is interpretable is to evaluate the coherence of the topic. The human topic ranking is the gold standard for assessing coherence, but it is pricy. In the current experiment, we implement a particular case of topic coherence using Eq. 2.

2 $$\:Cv=\frac{2}{N\left(N-1\right)}{\sum\:}_{i=1}^{N-1}\:{\sum\:}_{j=i+1}^{N}sim\left(\text{w}i,\text{w}j\right)\:$$

Where, N is the total number of terms in the top-N list for the given topic, sim(w*i*, w*j*) is the Jaccard similarity between the sets of documents containing words w*i* and w*j*.

**Fig. 5:**
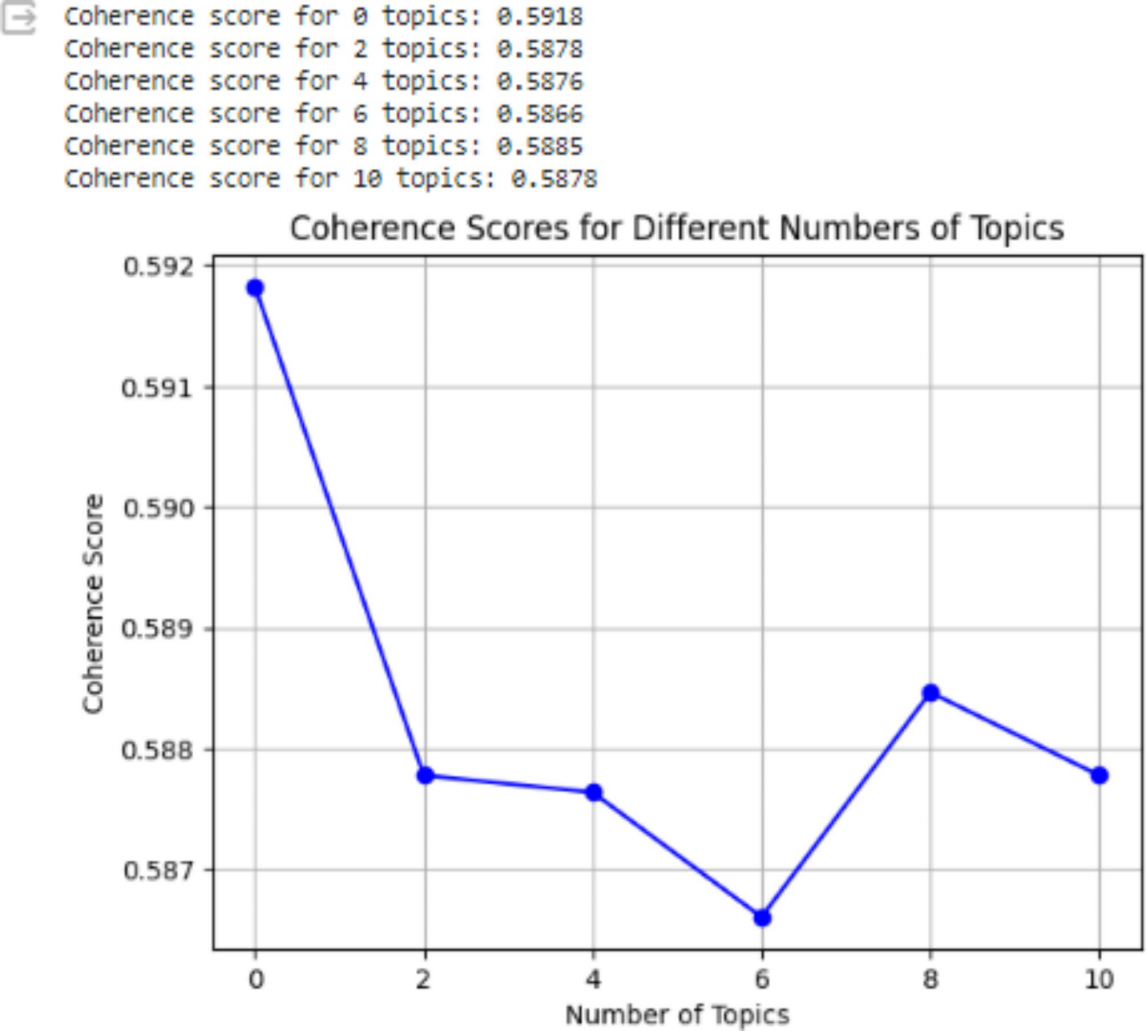
Topic model Coherence values using LDA algorithms.

In our experiment, topic modeling is employed for topics 2, 4, 6, 8, 10, and the coherence score is nearly the same for each generated topic as observed, ranging from 0.5918 to 0.5878. In this work, extracted topics are well interpretable at topic 10 by considering coherence values and human expert judgment. The interpretability of the topics in our methodologies coherence values is illustrated in Fig. 5.

In this work, we assess our model results based on interpretability quality criteria through human inspection and topic coherence metrics. In human judgment, the decisions of each resulting topic are manually recorded to articulate whether the topic provides a meaning or not.

Now, in order to mine further specific medical information, the technique is needed to advance retrieval performance. Finding the relevant document within this collection of documents in response to the user query is known as information retrieval^46^. As the information retrieval wants to address ambiguous knowledge, the exact processing techniques are not appropriate^47^. The probabilistic models are more suitable for these techniques. Within this model, relations are provided with probability weights corresponding to the significance of the document. This weight reflects different ranks of relevance. The results of the current IR schemes are regularly sorted lists of the documents where the top outcomes probabilities are more expected to be relevant with the search index according to the systems. In some methods, the users can judge the returned results and tell the methods which ones are applicable for them. The method then resorts to the result set. Documents that have many of the arguments present in them are ranked as having a higher probability value^48^. These relevance response processes are known to greatly advance IR performance. As a search key from the corpus, we can use document ID, document name, or document keywords. In our experiment as search queries, we use document identity numbers to retrieve respected topic information through the LDA model. As a consequence, the model tries to retrieve relevant topic information from a query entered by the user. As per the LDA model assumptions, every document is a mixture of topics. The model can also be able to visualize this topic mix and dominant topics in each document. For instance, when the user provides document ID 1018 to the developed model, which contains **“**gurra keessa waraana, ciniina**”** are displayed as the topic weight distributions. This means the model finds information about the given document ID from our corpus. Topic mix for sample documents is as presented in Table 5.

**Table 5:** Presents the information retrieved from our corpus based on document ID.

Document ID	Dominant topic ID	Different topic contribution percentage in a document
769	Topic 1	Topic 1(**0.8050**), Topic 5(0.1050), Topic 0(0.0550), Topic 6(0.0050), Topic 4(0.0050), Topic 2(0.0050), Topic 3(0.0050), Topic 7(0.0050), Topic 8(0.0050), Topic 9(0.0050),
1018	Topic 6	Topic 6(**0.7101**), Topic 2(0.1899), Topic 4(0.0125), Topic 9(0.0125), Topic 0(0.0125), Topic 7(0.0125), Topic 1(0.0125), Topic 3(0.0125), Topic 5(0.0125), Topic 8(0.0125)
1071	Topic 4	Topic 4(**0.7750**), Topic 2(0.0250), Topic 9 (0.0250), Topic 6(0.0250), Topic 5(0.0250), Topic 3(0.0250), Topic 0(0.0250), Topic 1(0.0250), Topic 7(0.0250), Topic 8(0.0250)

The bold value is representing the high probability contribution values of topics by applying LDA model.

###  Document classification analysis and evaluation

As we mentioned in the above dominant topic extraction section, each document was allocated to one category based on the highest topic distribution weight. This is important when we execute topic models on the same dataset again and again to find the best k number of topics. We trained the LDA model to extract the topics; then after, we required to go further with the health document classification in Afaan Oromo with the model. We have gone through document categorization experiments according to the topics. Once a topic classifier is trained using the LDA models, we need to test the topic distribution in the documents.

We have randomly selected 600 (20%) of documents as a test set from the total datasets. After performing the experiment, we manually labeled this test document with a specific label name for every document. We compared the manually labeled test documents with the results of LDA-labeled documents. We used common metrics, namely accuracy and F1 score. Then, we computed the effectiveness of the LDA model output in order to realize how the model applied is good enough for the rest of the whole dataset. Although our models classify most of the problem statements accurately and give the correct class labels for the test documents, we also analyzed a few examples where our model could not give accurate results due to a few constraints of our dataset.

In the context of topic modeling and NLP, the main challenge of polysemy (words with multiple meanings) and homonymy (words that are spelled the same but have different meanings) can significantly impact the interpretability and accuracy of the model. For instance, from our dataset, the word **“Dhukkuba”** can mean “disease” or “pain”. In a medical-related document, it could refer to a specific illness, general pain, or symptoms, depending on its context. The word **“Nyaata”** can mean “food” (noun) or “eats” (verb). The context of usage will determine whether it refers to the food itself or to the act of eating. This can make it difficult for the model to clearly distinguish between the topics. However, our model shows promise results when we evaluate our test documents. From this experiment, we obtained 79.17% accuracy and 79.66% F1 score for the LDA model results that we tested with the similar training set. These metrics are promising, especially in the context of unsupervised topic modeling where such performance levels are often considered robust. In this framework, these scores show the model’s ability to generate coherent topics that align well with the underlying structure of the data.

### Medical specialist recommendation to patients

**Fig. 6:**
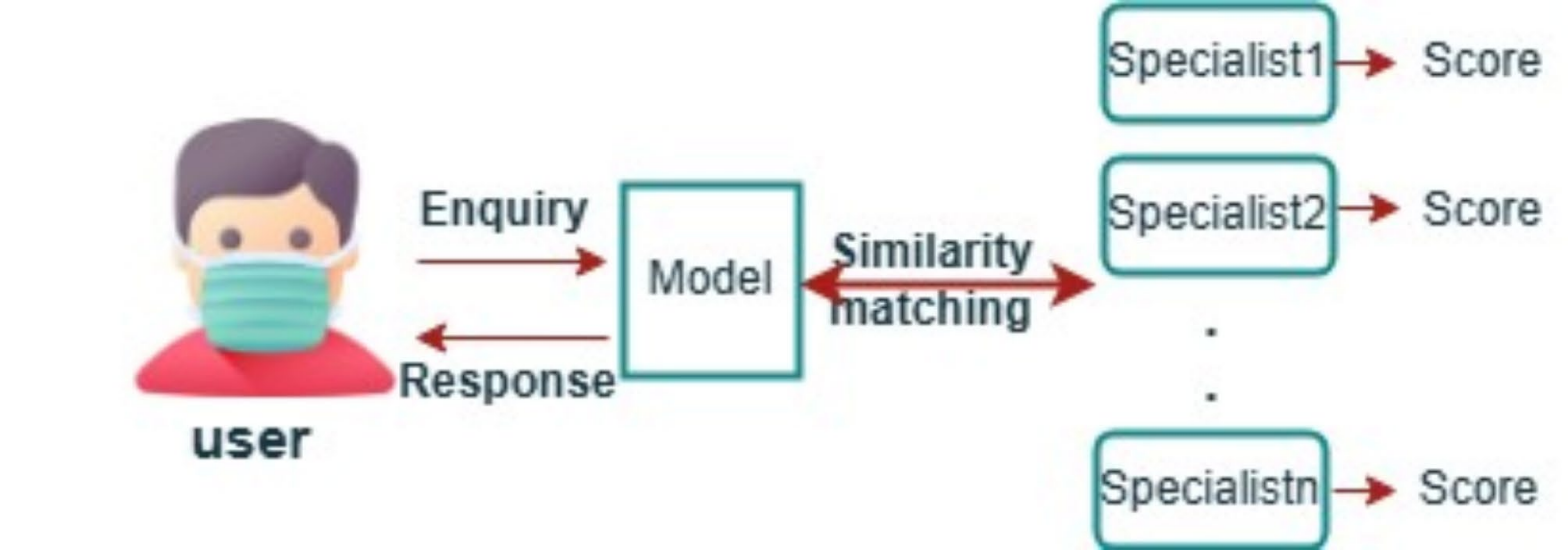
Medical specialist prediction model architecture.

In this work, we present a recommender method that helps the users find solutions for their health conditions. Individuals spend more time and need various efforts to search doctors and medical departments according to the specialties provided. For this task, we have successfully created the corpus, the topics, and the documents associated with specific labels. The proposed method provides efficient medical specialists recommendations to fill this gab. The model takes an input inquiry from the user. This taken input inquiry is considered, and it gets sent into our model to the most satisfying output as the medical specialists and from that we generate and give out the most probable suggestions that can be used by the patient based on the most similar weight. The model will choose the highly predictive similar documents for personalized recommendation to the target users. According to our integrated systems, it recommends the documents with the highest predicted score value to the users, as presented in Fig. 6. The model result is deemed accurate if the patient’s consultation query falls within the disease class in which the suggested medical professionals are highly skilled.

**Fig. 7:**
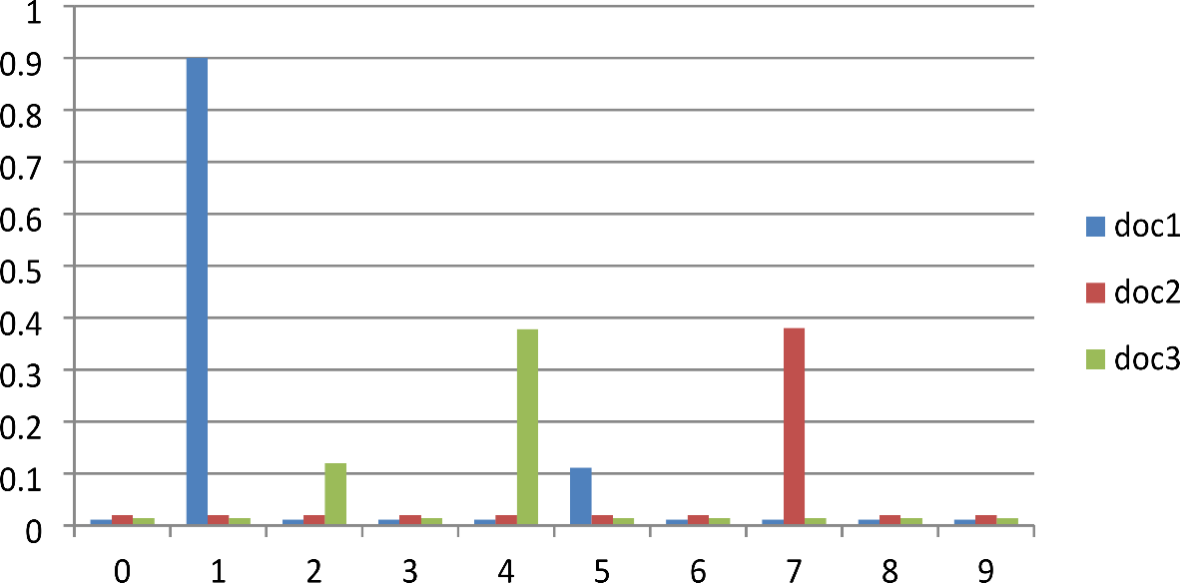
Graphical representations of documents topic distribution of unseen data using our model **(Note**: Nervous disease = 0, Gynecology = 1, Mental illness = 2, Eye disease = 3, Ear disease and disorders = 4, Internal disease = 5, Chronic disease = 6, Skin disease = 7, Orthopedic conditions = 8, Dental disease = 9).

Finally, we use our model to make predictions of the suitable medical specialists for new documents or patent queries provided by the users. In this experiment, the output of the LDA results, the topics and topic probabilities associated with trained documents, would result in labeled documents that would finally be useful to classify unseen documents. Figure 7 illustrates the document-word distribution weights for the ten topic or specialists departments. Each document under each topic is assigned a weight, with the higher ranking documents having greater weights. Based on the higher ranking, the model will recommend the highest topic name for each inserted document, as presented in Table 6. The users can also view the model generated response for the provided unseen documents.

**Table 6:** Presents our model topic labels prediction of unseen documents of the AOHD.

Document	Input enquires	Predicted results
doc1	xuriin seeran natti hin dhufu yeroo tokko tokko ji’a darbee dhufa yeroo biraa ture dhufa	*Gynecology*
doc2	qaama koo irra deeme na madeessa, na hooksisa	*Skin disease*
doc3	gurra koo keessa na waca fagoo irraa sagalee hin dhaga’u	*Ear disease and disorders*

## Discussion

In this article, we have presented how it is possible to discover hidden topics of electronic health documents in Afaan Oromo and make them suitable for information retrieval and the classification process, which are used in very important tasks to accomplish healthcare organizations goals. As noted above, we used the LDA, which is a very popular technique used to discover hidden topics in a group of documents. It provides a straightforward probabilistic framework that is easy to interpret and understand. One of the advantages of LDA in this work is that the model does not require preliminary training^49^. As a result, it is possible to obtain well-interpretable documents regardless of the length of the texts. The other advantage is that the LDA model is independent of specific languages that work with the bag-of-word model, which disregards the order of the words. Each textual document is viewed as a mixture of topics, and the extracted topics are a mixture of words. In this study we consider two foremost LDA parameters. These parameters are number of topics(n_topic) and the number of words per topic (n_topic_word). We selected n_topic = 10 and n_topic_word = 10 since they outperform slightly results than the other values. However, finding the optimal values can require running a greedy search. For this experiment, the default value of 0.1 is assigned to both the alpha, which states a dirichlet prior on the topic distributions per document, and beta parameter, which describes the prior on the multinomial distributions of words per topic. So, the results of LDA are easy to interpret, providing a clear understanding of which topics are predominant in a set of documents and the construct distributions associated with each topic.

Since our current work is the first attempt with these specific low-resource languages and health domains, we are unable to compare our experimental result to other existing research works. The originality of our research presents both an opportunity and a challenge. On the one hand, it unlocks up new possibilities for exploration and sets a foundational benchmark for future studies in this linguistic context and related to health documents. On the other hand, the lack of existing literature and comparative studies in these languages means we do not have prior research to validate our findings against or to draw direct comparisons from. This unique concept underlines the importance and pioneering nature of our study, highlighting its potential to contribute significantly to the academic research and practical understanding of topic modeling techniques of these languages in the context of our research area.

## Conclusion and future work

This research focuses on topic modeling for the electronic health documents in Afaan Oromo using the latent dirichlet allocation (LDA) as an algorithm. This work’s novel contribution is the effective categorization and organization of the documents into more precise classes using the topic modeling techniques. The dataset collected contains health related electronic documents without labels in this experiment. Hence, our model does not require labeled documents and hence minimizes the cognitive load of the annotators. The dataset passed through different preprocessing levels to make it suitable for training the model. We experimentally demonstrate the model with the cleaned documents. After obtaining extracted topics and creating weight values for each keyword in the provided documents, then the model was employed to anticipate the most relevant test document topics. We used the applications of topic modeling for information retrieval and to find medical specialists who are suitable for the patient’s based information provided. In this work, the model involves only the patient’s description of their conditions and is not required to give any other user input. As a result, the LDA model gave the promised value of 79.17% accuracy and 79.66% F1 score over topics for test documents of the dataset.

Despite focusing on Afaan Oromo health document topic modeling using LDA, limitations emerge from resource restrictions and contextual meanings of the terms. Due to this, the LDA may have trouble with polysemy and high correlation between topics, which can influence its accuracy and interpretability in the datasets. To overcome these issues, future work should explore LDA2vec techniques combining models word embedding to capture the contextual meanings of each word^42^. LDA2Vec practices semantic embedding to find topics, making it more robust in identifying the polysemy (words with multiple meanings) and words context.

## Data Availability

The data and material used during the current study will be available from the corresponding author upon request and available on the public repository: Etanafik/Topic-Modeling-for-Medical-domain.
